# Management of hidradenitis suppurativa in the inpatient setting: a clinical guide

**DOI:** 10.1007/s00403-024-03622-9

**Published:** 2025-01-08

**Authors:** Narges Maskan Bermudez, Scott A. Elman, Robert S. Kirsner, Hadar Lev-Tov

**Affiliations:** Dr Philip Frost Department of Dermatology and Cutaneous Surgery, University of Miami Miller School of Medicine, 1600 NW 10th Ave RMSB 2023A, Miami, FL USA

**Keywords:** Hidradenitits suppurativa, Inpatient management, Admission criteria, Flare, Rescue therapy

## Abstract

Hidradenitis suppurativa (HS) is a chronic inflammatory disease that affects the axilla, inframammary folds, buttocks, inner thighs, and anogenital regions. Patients with moderate to severe HS often seek care in the emergency department and may require hospitalization; however, the lack of clear admission criteria and management guidelines presents significant challenges. To address these issues, we conducted a clinical review aimed at hospitalists and consulting dermatologists considering hospital admission for patients experiencing severe HS flares. Admission offers a unique opportunity to optimize care through specialized consultations, pain management, rescue therapy, and surgical planning. Furthermore, multimodal treatments, particularly biologics, are needed to achieve clinical remission in severe disease. Streamlining care during hospitalization for the early initiation of these therapies can significantly improve flare management and overall clinical outcomes for HS patients. This review aims to improve care for HS by providing clear and comprehensive guidance on its management in the inpatient setting.

## Introduction

Hidradenitis suppurativa (HS) is a chronic inflammatory disease that affects the axilla, inframammary region, buttocks, inner thighs, and anogenital areas. Clinical presentations range from mild papules and pustules to severe abscesses, tunneling, and scarring [1, 2]. HS disproportionally affects women, young adults, and individuals of African descent [3], with risk factors such as genetics, metabolic syndrome, smoking, and obesity [4–7]. Some patients may also have a family history of HS or related follicular occlusion disorders [8]. Diagnosis typically occurs 7–10 years after onset, most commonly during the second or third decade of life [9].

This diagnostic delay often leads to emergency department (ED) visits during acute flares. Misdiagnosis, particularly in moderate to severe cases, can result in inadequate management and frequent ED visits. Patients from lower socioeconomic backgrounds may face additional challenges, including limited access to care, higher disease burden, and increased comorbidities [10]. As a result, patients with moderate to severe HS may require hospitalization during severe flares for symptom management and advanced care. However, there are no established inpatient protocols or admission criteria for HS patients.

This comprehensive literature review examines these gaps in care and opportunities for improving inpatient management of HS. It serves as a resource for hospitalists, consulting dermatologists, other healthcare providers, and allied health professionals who may encounter HS patients in the inpatient setting. We offer insights into patient selection for admission and summarize recent findings on management strategies and therapeutic options.

## Methods

We conducted a comprehensive literature search on PubMed/MEDLINE to identify studies on HS management, particularly in inpatient care and therapies. The search included articles published up to April 2024, using a combination of MeSH terms and keywords such as "hidradenitis suppurativa," "inpatient care," "hospitalization," "rescue therapy," "management," and "treatment." We reviewed all relevant, peer-reviewed articles, excluding non-English studies and those unrelated to HS management.

## Comprehensive management of hidradenitis suppurativa

### Treatment challenges in hidradenitis suppurativa

Effective management of HS requires a multidisciplinary approach that integrates medical, surgical, and psychosocial therapies to achieve remission. Current medical interventions include lifestyle adjustments, topical treatments, oral medications, intravenous (IV) antibiotics, and biologic therapy [11, 12]. Among these, biologic therapies have significantly transformed HS management, particularly the FDA approval of adalimumab, secukinumab, and bimekizumab [13–19]. Studies show that early initiation of biologics, when combined with surgical interventions, can lead to better outcomes [13, 15–17, 20, 21]. However, high costs and the need for prior authorization can create barriers to access, limiting optimal patient care [14].

### Optimizing medical and surgical management

For surgical candidates, available options include wide local excisions, tunnel deroofing, incision and drainage, and laser therapy [22]. Optimizing medical management with hormonal, antibiotic, or biologic therapies is needed for improved outcomes. Additionally, addressing comorbid conditions can further improve disease control and reduce the risk of recurrence [23]. It is also crucial to integrate psychosocial care, as many HS patients experience anxiety and depression stemming from the chronic nature of their condition [24–26]. Therefore, treatment guidelines should prioritize managing active disease, preventing new flares, and addressing psychosocial barriers.

### Healthcare utilization patterns and access challenges

Patients with HS utilize ED and inpatient services more frequently than those with other skin conditions [27, 28]. The typical demographic includes low-income women aged 18–39 with Medicaid insurance. Notably, ED visits increase during the summer months, particularly in the southern U.S. regions [29, 30]. Since the 1990s, hospitalization rates for HS have risen by 60%, with approximately 10.2% of HS-related ED visits leading to hospitalization between 2013 and 2015 [29, 30]. One study revealed that 17.2% of patients revisit the ED within 30 days of their initial visit, while only 2.4% attend dermatology follow-ups. Over six months, 34% of patients return to the ED for HS care, compared to just 6.8% who received dermatology follow-ups, highlighting frequent ED usage and difficulties in accessing consistent dermatologic care [31].

**Fig. 1:**
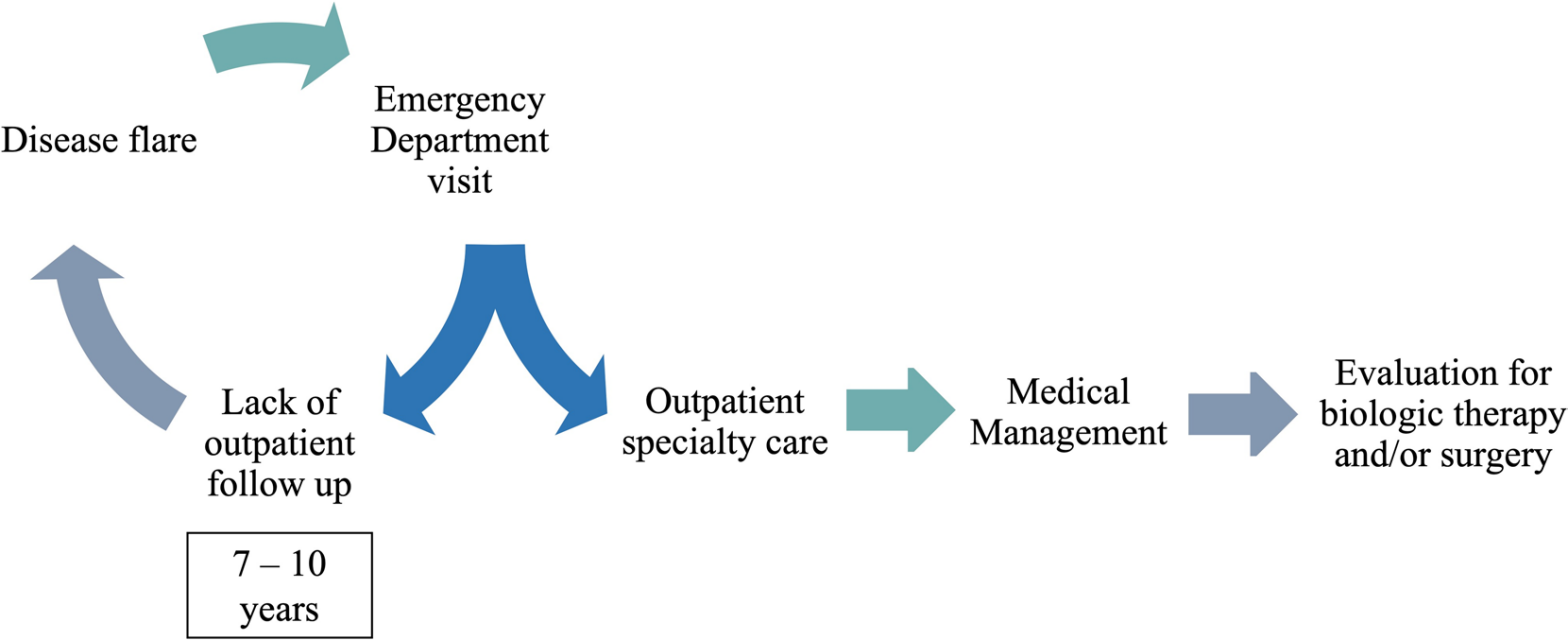
Delay in diagnosis and repeated ED visits in patients with HS. Early intervention can allow specialty follow-up care and appropriate medical management

These patterns contribute to significant healthcare costs, primarily from recurrent ED visits, inpatient admissions, and delayed diagnoses [10, 27, 32]. Consequently, a problematic scenario arises: patients with complicated disease often do not receive optimal care, and even when patients access costly care settings, management often remains suboptimal, leading to disease flares soon after discharge (Fig. 1).

### Recommendations for admission

**Fig. 2:**
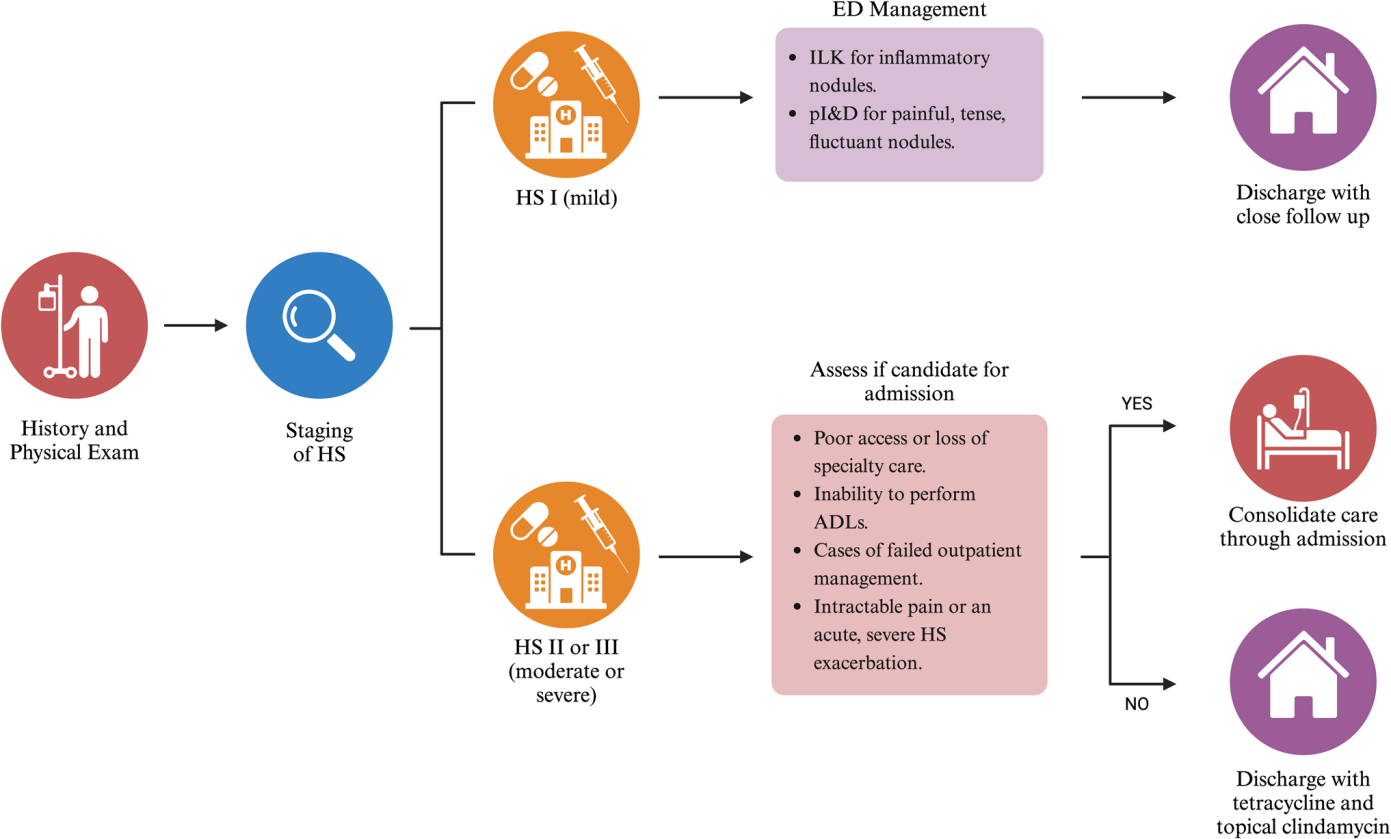
Proposed guidelines for disposition. Patients with moderate to severe HS who meet admission criteria can benefit from consolidation of care through the inpatient setting

This review outlines a comprehensive approach to determining whether a patient with HS is a candidate for admission (Fig. 2). Patients with mild disease can usually be managed as an outpatient, while those with painful, fluctuating abscesses may benefit from modified punch incision and drainage (pI&D) before ED discharge [33].

For moderate to severe cases, several factors can influence admission decisions, including access to follow-up care, availability of medical therapies, self-care capacity, activities of daily living (ADLs), and support systems. Admission may be warranted for patients who have not responded to outpatient management, experience intractable pain, have difficulty accessing specialist care, or suffer from acute exacerbations. In such cases, admission to a medical service is recommended for further evaluation.

## Approach to inpatient management

**Fig. 3:**
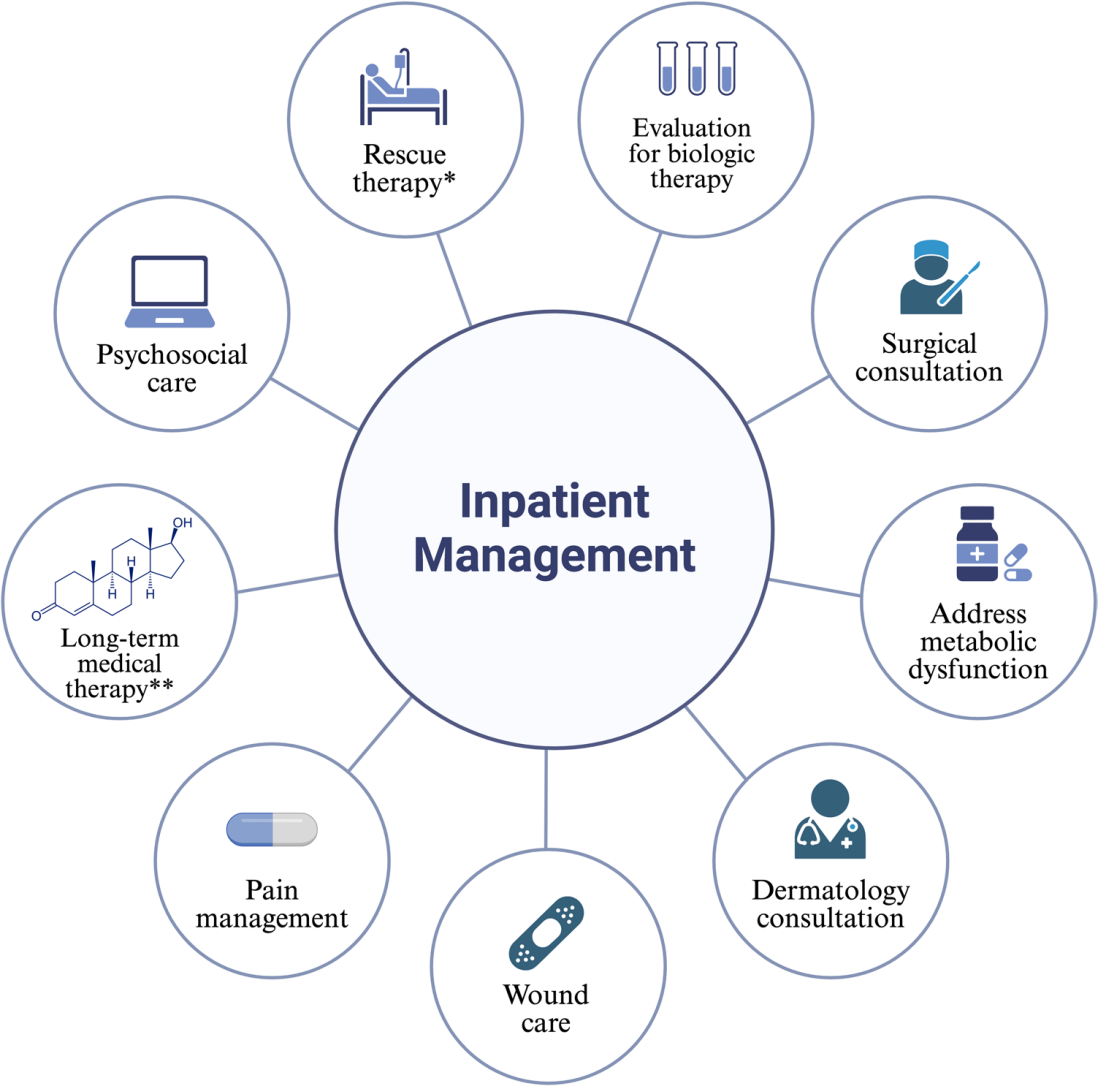
Approach to inpatient management. *Rescue therapy can include IV ertapenem and/or steroids. **Long-term therapy includes hormonal therapy (i.e. spironolactone), anti-inflammatories, antibiotics, and/or biologics

This section outlines the inpatient management of HS, covering diagnosis, staging, laboratory tests, imaging, inpatient therapies, specialty consultations, and discharge planning (Fig. 3). Dermatology consultations should be initiated for patients with severe disease, recurrent admissions, or ineffective outpatient treatments to ensure continuity of care. Additional specialist consultations, including surgery, pain management, wound care, nutrition, and infectious disease (ID), may be necessary for complex cases requiring long-term antibiotic therapy.

Managing comorbid conditions such as inflammatory bowel disease, cardiovascular disease, diabetes, and obesity is crucial, as these can significantly impact HS activity [5, 7, 23, 34, 35]. Acne, dissecting cellulitis of the scalp, pilonidal cysts, depression, anxiety, tobacco use, and polycystic ovary syndrome are also associated with HS and can be screened for during admission however, the benefit may be tangential in the acute care setting [34]. Social work services can help coordinate care, address financial or insurance barriers, and assist with transportation or housing needs, and this may represent a key benefit to inpatient admission. Though more research is needed, inpatient care may improve long-term outcomes for HS patients.

### Diagnosis and staging of HS

Diagnosing HS requires a thorough evaluation, including a detailed medical history and physical examination, focusing on recurrent or progressive lesions (nodules, abscesses, tunnels, scarring) in intertriginous areas [12]. The Hurley disease staging system, although limited, is simple and frequently used across specialties to characterize disease severity and guide therapeutic regimens. Hurley stage I is characterized by single or multiple nodules and abscesses without tunnels or scarring. Hurley stage II is characterized by recurrent widely separated nodules and abscesses with a limited number of tunnels and/or scarring. Hurley stage III is characterized by diffuse, multiple, or extensive nodules and abscesses with interconnecting sinus tracts and/or scarring [36].

### Laboratory studies

There are no specific laboratory tests for diagnosing HS. Sepsis is not typically caused by HS lesions. However, cultures may be warranted if primary infection or secondary cellulitis is suspected [37]. Systemic inflammatory response syndrome (SIRS) is rare but can occur in severe cases [37]. SIRS is defined by two or more of the following criteria [38]:

- Body temperature > 38 °C (100.4°F) or < 36 °C (96.8°F).
- Heart rate > 90 bpm.
- Respiratory rate > 20 breaths per minute or PaCO₂ < 32 mmHg.
- White blood cell count > 12,000/mm, < 4000/mm, or > 10% immature forms.

Although rare, clinicians should monitor for signs of systemic infection in advanced or complicated cases of HS (e.g. patients on immune-suppressing medications).

No laboratory tests can independently predict morbidity or mortality in HS; however, certain biomarkers can indicate disease severity and associated comorbidities. For example, elevated C-reactive protein (CRP) and erythrocyte sedimentation rate (ESR) levels indicate systemic inflammation and are often associated with more severe HS, though they are not specific to the condition [39–42]. Serum levels of interleukin 6 (IL-6) have also been investigated for correlation with disease severity and demonstrate moderate correlation, which may be useful to monitor treatment response [43–48]. Ultimately, the skin exam remains the best biomarker for severity and disease activity. Anemia is common in HS, and elevated hepcidin levels can help differentiate anemia of chronic disease from iron deficiency [49, 50]. Comorbidities such as diabetes, obesity, and metabolic syndrome also increase morbidity in HS patients [35, 51]. Screening for diabetes using hemoglobin A1C can aid in managing these comorbidities through lifestyle changes and medical interventions [34].

For patients with moderate to severe HS who are candidates for biologic therapy (discussed in Section “Biologic Therapy”), baseline tests can be performed during the inpatient stay, including a complete blood count (CBC), comprehensive metabolic panel (CMP), liver function tests, hepatitis panel, and screenings for latent tuberculosis [52].

### Imaging studies

Imaging studies should not be used for HS staging or diagnosis; however, they can be valuable in select cases where deeper involvement or complications are suspected. This includes situations with extensive lesions or concerns about fistulas or hidden abscesses, particularly near joints or bones [53]. Imaging is also useful in pre-surgical planning by mapping sinus tracts and abscesses to guide precise excisions [54–56]. It can provide diagnostic clarity in cases with atypical presentations or when alternative diagnoses like malignancy or infections are considered. While ultrasound is often used for real-time evaluation, MRI is better suited for assessing severe anogenital disease, fistulas, deep tissue involvement, or extensive disease [57–59].

### Procedures

pI&D should be reserved for patients seeking temporary symptomatic relief from tense, painful, fluctuant abscesses, as traditional I&D is associated with a 100% recurrence rate [60]. Patients should be informed that this relief is temporary. An alternative for managing inflammatory nodules is intralesional triamcinolone (10 mg/mL, 0.7–2 mL per lesion), which many patients report improves pain and inflammation within 48 h [61].

### Antibiotics

HS has an inflammatory rather than infectious etiology, but certain antibiotics have significantly improved disease activity. It is unclear if antibiotics work by exerting anti-inflammatory effects or by disrupting the microbiome that induces inflammation. North American guidelines recommend topical clindamycin lotion and oral tetracyclines as first-line treatments for mild to moderate HS, followed by oral clindamycin and rifampin as second-line treatments for mild to moderate disease or as first-line or adjunctive treatments for severe disease. For moderate to severe HS, a combination of moxifloxacin, metronidazole, and rifampin is recommended as second or third-line therapy [11].

In select cases, IV antibiotics may be used as rescue therapy, providing a bridge to long-term maintenance. This will be discussed further in the following section on rescue therapy.

### Rescue therapy

A key advantage of hospital admission is the ability to initiate aggressive treatment for HS with IV rescue therapy. A promising approach is a 6-week regimen of ertapenem, which has shown clinical remission and significant improvements in quality of life for patients with advanced HS [11, 45, 62–64]. Ertapenem can be started in the inpatient setting as a bridge to new therapies or to allow existing ones to take effect. After inserting a peripherally inserted central catheter (PICC), social workers can coordinate home healthcare for daily infusions. Its once-daily dosing is more practical than meropenem, which requires infusions every eight hours [65]. Additionally, some evidence suggests that longer courses of ertapenem, up to 12–13 weeks, may result in further improvements in clinical severity and pain [45]. Emerging data on intramuscular administration may also simplify its use [66]. However, patients often relapse within weeks of discontinuing ertapenem, so it should be reserved for severe Hurley stage III flares when other treatments have failed, with a clear transition plan to surgery or biologic therapies. Due to the risks of antibiotic resistance and Clostridium difficile infection associated with IV ertapenem [67], its overuse should be avoided, and strong antibiotic stewardship should be maintained. Consulting ID specialists is beneficial, especially for patients requiring prolonged or multiple courses of antibiotics.

IV steroids (0.5 mg/kg to 1 mg/kg) can be administered in combination with IV ertapenem or as monotherapy, particularly after recent ertapenem courses. Upon discharge, patients should taper to oral steroids [68]. Sirolimus and linezolid have also been studied as rescue therapies for HS, but data on their efficacy remains limited [69, 70].

### Biologic therapy

Patients admitted with HS should be evaluated for biologic therapy, especially those who have not responded to standard treatments or have severe disease impacting their quality of life. FDA-approved biologic options include adalimumab, secukinumab, and bimekizumab, while infliximab is frequently used off-label [18, 52, 71–73]. An Italian study identified a 'window of opportunity' for early biologic intervention, demonstrating that initiating biologics like adalimumab early leads to better outcomes, particularly when used before the development of permanent scarring and sinus tracts [16]. Biologics have also shown improved outcomes when combined with surgery [15]. This combination can improve overall disease control and may reduce the need for more extensive surgical procedures in the future.

To facilitate outpatient initiation of biologic therapy, dermatology coordination is required, and necessary lab work can be completed during the hospital stay. This workup includes a CBC, CMP, liver function tests, and screenings for hepatitis and latent tuberculosis [52].

### Pain management

HS pain involves both neuropathic and nociceptive pathways [12, 74]. Nociceptive pain arises from acute tissue injury, while neuropathic pain stems from chronic inflammation, often leading to hyperalgesia due to central sensitization [75, 76]. This can explain why some patients continue to experience pain even after inflammation resolves, often necessitating higher doses or more potent analgesics [76, 77].

A multimodal approach to pain management is recommended, with first-line therapies including topical analgesics, acetaminophen, and nonsteroidal anti-inflammatory drugs (NSAIDs) [12]. Anticonvulsants and selective serotonin reuptake inhibitors (SSRIs)/serotonin-norepinephrine reuptake inhibitors (SNRIs) may also help relieve neuropathic pain by modifying neural thresholds, making them potentially useful for the long-term management of HS. [78, 79]. Regular pain assessments are essential, with dosing and pharmacologic options summarized in Table 1 [12, 75]. Additionally, long-term disease control is key to reducing pain [12].

**Table 1:** Modified from Savage et al. "Pain Management in hidradenitis suppurativa: a proposed treatment algorithm"

Medication	Mechanism	Dosage*
Acute pain		
Acetaminophen [75, 80]	Inhibits central nervous system prostaglandin synthesis	325–650 mg orally every 4–6 h **Max dose:** 3000 mg/day
Lidocaine topical [75, 81]	Blocks neuronal Na + channels, reducing pain sensation	**Cream (4-5%):** apply to skin up to 3 to 4 times daily **Patch (4%):** frequency and duration vary. Refer to the manufacturer’s labeling for recommendations
Intralesional triamcinolone [75]	Decreases neutrophil extravasation and cytokine signaling	10–40 mg/mL; 0.7–2.0 mL injected per lesion Repeat every 1–3 months
NSAIDs [75, 82, 83]	Nonselective COX inhibitor, reduces prostaglandin-mediated pain	**Ibuprofen:** 400 mg every 4–6 h, Max dose: 2400 mg/day **Naproxen (immediate release):** 250 mg every 6–8 h **Max dose: **1000 mg/day **Naproxen (extended release):** 1 g once daily, can increase to 1.5 g/day during severe flare
Chronic pain		
Gabapentin [75, 84]	Binds α2δ subunit of voltage-dependent calcium channels, reducing neuropathic pain	**Day 1:** 300 mg once daily **Day 2:** 300 mg twice daily **Day 3: **300 mg three times daily Increase by 300 mg daily until reaching max tolerated dose **Max dose:** 3600 mg/day, divided TID
Pregabalin [75, 85]	Similar to gabapentin, binds α2δ subunit of calcium channels	**Initial dose:** 150 mg daily in divided doses Can increase to 300 mg daily after one week **Max dose:** 600 mg/day in divided doses
Duloxetine [75, 78, 79, 86]	Selective serotonin and norepinephrine reuptake inhibitor	*Consider in patients who failed gabapentin and pregabalin 60 mg once daily
Venlafaxine [75, 78, 79, 87]	Selective serotonin and norepinephrine reuptake inhibitor	*Consider in patients who failed gabapentin and pregabalin **Initial dose:** 75 mg once daily Increase by 75 mg each week until reaching max tolerated dose **Max dose:** 225 mg/day

*Dosing adjustments may be needed in cases of renal or liver impairment

Opioids should be reserved for short-term management of severe, acute pain that does not respond to first-line treatments, given their risks and potential for misuse. Tramadol is the preferred first-line opioid, with other options including codeine, hydrocodone, and morphine [75]. However, opioids are no more effective than opioid-sparing analgesics and carry significant risks of dependence [77, 88].

Given the complexity of managing HS-related pain and the risks associated with long-term opioid use, consulting a pain specialist during inpatient care can be beneficial, especially in cases of chronic pain requiring adjunct therapy [76–79].

### Wound care and dressings

Acute HS exacerbations often result in significant drainage, which can greatly impact patients’ quality of life. Dressing selection should be individualized based on factors such as wound depth, exudate amount, tunneling, location, and patient preference [89]. Foam dressings are widely used in hospital settings for their absorbency and cushioning properties, making them suitable for large, draining wounds. For highly exudative wounds, alginate dressings (derived from seaweed) and gelling fiber dressings are effective in absorbing large amounts of fluid and promoting healing [89].

While no single dressing type is ideal for all HS cases, a kit of foam, gelling fiber, hydrogel dressings, and tape may improve the quality of life for patients with persistent drainage issues [89–91]. Wound care consultations can be beneficial in complex cases, and arranging follow-up with a wound care center upon discharge is crucial to ensure patients receive appropriate dressing supplies and regular re-evaluation. Cost is also an important consideration, with foam and alginate dressings being more affordable, whereas gelling fibers offer better exudate management.

### Psychosocial care

HS imposes a significant psychosocial burden, leading to reduced quality of life for many patients. Studies indicate that depression and anxiety scores correlate with disease severity, emphasizing the importance of addressing these comorbidities in the inpatient setting [92]. Psychological or psychiatric consultations should be arranged as part of the inpatient care process. Medical management of HS, particularly with therapies aimed at reducing disease severity, can also improve psychosocial outcomes. Additionally, psychosocial support may include counseling on coping strategies and improving patient adherence to treatment plans.

### Pregnancy and pediatric cases

Inpatient management of HS in pregnant and pediatric patients has not been extensively studied. Studies suggest that most pregnant patients with HS experience stable or worsening symptoms during pregnancy, with more than half reporting flares postpartum [93]. Topical clindamycin is generally considered safe during pregnancy [94]. While oral clindamycin, often used in combination with rifampin in non-pregnant patients, is safe in pregnancy, rifampin should only be used in special circumstances [95, 96]. Rescue therapies, such as ertapenem and corticosteroids, can be utilized during pregnancy, but long-term or high-dose corticosteroid use requires careful consideration and consultation with an obstetrician [93, 97, 98]. For moderate to severe HS, intralesional triamcinolone can provide symptomatic relief for acute inflammatory nodules during pregnancy [99].

Tetracyclines are contraindicated in both pregnant patients and children under nine years old due to their impact on bone and teeth development [100]. Regarding biologics, adalimumab increasingly crosses the placenta with pregnancy progression and is associated with an increased risk of birth defects [52]. The data on secukinumab and bimekizumab during pregnancy is limited, and its safety profile remains uncertain [18, 73]. Biologics should generally be discontinued by the third trimester to minimize placental transfer risks. Therefore, close collaboration with obstetricians and pediatricians, especially for nursing mothers, is advised. We note, however, that uncontrolled, severe HS (i.e., the population likely to be admitted) may be an independent risk factor for poor outcomes for both the mother (e.g., gestational diabetes mellitus, cesarean section) and the fetus (e.g., congenital defects, spontaneous abortion) [101–103]. Therefore, in the management of pregnant patients, all interventions should be discussed with the mother to arrive at an informed consent to treatment.

## Discharge planning

Discharge planning for HS patients is critical to ensure continuity of care and effective long-term management. Dermatologists should work closely with the primary team to facilitate a safe and comprehensive discharge plan. For patients on IV ertapenem, social work planning should begin early to arrange home-based administration of infusions. For non-pregnant female patients, hormonal therapies such as spironolactone should be considered, as they may help control disease post-discharge. If the patient is not on IV ertapenem, oral antibiotics, particularly tetracyclines for their anti-inflammatory properties, can be prescribed along with topical clindamycin [11, 104–106]. Table 2 summarizes the specific prescribing information for topical, oral, and biologic therapies.

**Table 2:** Prescribing information for discharge planning in patients with HS

Medication	Dosage	Route	Duration	Special considerations
Clindamycin topical [11, 106]	Apply twice daily to affected areas	Topical	3–6 months or as needed	Continue use during flares. Educate on antibiotic resistance
Doxycycline [11, 104, 105]	100 mg twice daily	Oral	3–6 months, can be extended if needed	Monitor for photosensitivity, GI side effects
Clindamycin + Rifampin [107, 108]	300 mg of each twice daily	Oral	10–12 weeks	Continue if clinically improving; monitor for liver function
Spironolactone [109, 110]	50–100 mg daily	Oral	Long-term therapy	Caution in females of childbearing age
Ertapenem [11, 54–57]	1 g daily	IV or IM	Up to 6 weeks	Only used in select cases as a rescue therapy Review closely side effect profile
Biologic therapies				
Adalimumab [52]	160 mg initial, then 80 mg at week 2, then 40 mg weekly	Subcutaneous injection	Long-term, reassess at 12 weeks	Ensure insurance coverage, monitor for infections, injection technique
Secukinumab [18]	300 mg at weeks 0, 1, 2, 3, 4, then every 4 weeks	Subcutaneous injection	Long-term, reassess after 16 weeks	Ensure insurance coverage, monitor for infections, injection technique
Bimekizumab [73]	320 mg by at week 0, 2, 4, 6, 8, 10, 12, 14 and 16, then every 4	Subcutaneous injection	Long-term, reassess after 16 weeks	Ensure insurance coverage, monitor for infections, injection technique.

This table is not a comprehensive list of all available therapies for HS

Lifestyle modifications, including weight loss counseling, exercise, and smoking cessation (if applicable), are essential for long-term HS control. Ensuring follow-up with a wound care center is crucial to address ongoing wound care needs and maintain a steady supply of appropriate dressings.

Close dermatology follow-up within one week of discharge is recommended to assess treatment progress and initiate biologic therapy if appropriate. Patients should also be referred to surgery for further planning, with a timeline for surgical interventions once they are medically optimized.

## Conclusion

This review highlights the importance of optimizing inpatient admission to consolidate HS care through a team-based approach and significantly improve outcomes. Access to various specialty consultations, pain management, rescue therapy, evaluation for biologic therapy, and surgical planning facilitates prompt treatment for people with moderate to severe HS. Notably, prospective controlled data about HS management in the inpatient setting is very limited, and therefore, we acknowledge the limitations of our management suggestions. Although further studies are needed, the inpatient setting can be instrumental in optimizing care for these patients and initiating long-term dermatology care. Therefore, dermatologists, hospitalists, and all relevant healthcare providers should consider admission as a vital part of their treatment strategy for severely affected patients.

## Data Availability

No datasets were generated or analysed during the current study.
